# Association between Occupational Noise and Vibration Exposure and Insomnia among Workers in Korea

**DOI:** 10.3390/life10040046

**Published:** 2020-04-22

**Authors:** Fatima Nari, Yun Kyung Kim, Soo Hyun Kang, Eun-Cheol Park, Sung-In Jang

**Affiliations:** 1 Department of Public Health, Graduate School, Yonsei University, Seoul 03722, Korea; fatima@yuhs.ac (F.N.); hykh51128@yuhs.ac (Y.K.K.); kshyun@yuhs.ac (S.H.K.); 2 Institute of Health Services Research, Yonsei University, Seoul 03722, Korea; ecpark@yuhs.ac; 3 Department of Preventive Medicine, Yonsei University College of Medicine, 50 Yonsei-ro, Seodaemun-gu, Seoul 03722, Korea

**Keywords:** sleep disorders, insomnia, disorders of initiating and maintaining sleep, occupational noise exposure, occupational vibration exposure, occupational health

## Abstract

**Background:** The effect of noise and vibration exposure on disturbed sleep has been investigated in the past. However, this study was carried out to investigate the relationship between workplace noise and vibration exposure with insomnia amongst representative Korean workers, both simultaneously and separately. **Methods:** Our research analyzed an overall population of 30,837 workers aged 15 years or older using data derived from the 5th Korean Working Conditions Survey (KWCS) conducted in 2017. Chi-squared tests and logistic regression were performed to investigate baseline characteristics and to quantify the association between workplace exposure to noise and vibration with insomnia. Relative excess risk due to interaction (RERI), attributable proportion (AP), and synergy index (S) were calculated to measure interactions between simultaneous noise and vibration exposure with insomnia. **Results:** The prevalence of those who reported insomnia was 18.3% of the general population. Among men and women, insomnia in those who were exposed to noise only was 13.9% and 18.3%, respectively, and in those who were exposed to vibration only, it was 23.9% in males and 26.4% in females. Insomnia in those who were exposed to both noise and vibration simultaneously was 20.5% and 41.2% in men and women, respectively. The odds ratio (OR) of insomnia due to noise exposure was 1.10 and 1.07 in men and women, respectively. OR of vibration exposure was 1.84 in men and 1.58 in women. For noise plus vibration exposure OR was 1.83 in men and 3.14 in female workers, where the synergistic effect of noise and vibration exposure could be seen. The association between the varying degree of simultaneous noise plus vibration exposure with insomnia showed a dose–response relationship. The interaction measures showed a synergistic effect of simultaneous exposure in women but not in men. Conclusion: Our study revealed an association between occupational noise and vibration exposure and insomnia, both individually and simultaneously. Additional studies and research are required to further comprehend this relationship.

## 1. Introduction

Over the past few decades, an emphasis has been placed on investigating the effects of different occupational risk hazards on the safety and wellbeing of workers. Exposure to environmental stressors such as noise and vibration and their detrimental effects on health and diseases have been explored frequently in other studies. Some of these effects include, but are not limited to, physiological conditions such as hearing loss [1], cardiac and vascular problems [2,3], and elevated blood pressure [4,5], as well as nervous system-related disorders such as stress, annoyance [6], headache/eye strain [7], fatigue [8], and perhaps the most prominent nervous system issue—insomnia/sleep disturbance [9,10,11].

Sleep is essential for regeneration of the body [12], and therefore, poor quality of sleep or disturbed sleep can be of significance for immediate and long-term health [13]. Insomnia may be of qualitative or quantitative nature and is characterized by difficulty of falling asleep and frequent awakenings leading to unsatisfying or unrefreshing sleep [11,14]. Disturbed sleep can result in consequential effects such as daytime sleepiness [15], fatigue, and reduced mental and cognitive function [16,17]. Negative outcomes of insufficient sleep in the workplace include increased workplace aggression [18], decreased work productivity, absenteeism, and a rise in the number of occupational accidents and injuries [19,20].

The adverse effects of noise on sleep have been thoroughly researched, and there are existing guidelines regarding noise exposure consequences [21], but the mechanism of how vibration influences sleep is still unclear. Even more lacking is knowledge relating to the synergistic effect of combined noise and vibration exposure on insomnia. Investigations regarding occupational noise and vibration exposures can be quite challenging due to the fact that extra-occupational sources of noise and vibration are quite numerous [22,23,24]. Therefore, it is difficult to identify risks associated with those two factors.

Our objective in this study was to investigate the association between noise and vibration exposure and insomnia both individually and together. This set it apart from prior studies that concentrated on the relationship between insomnia and each of the two risk factors separately. In addition, we aimed to find out whether the combined effect of occupational noise and vibration exposures was synergistic and to compare the two factors and determine which one has a larger influence on insomnia.

## 2. Results

The general characteristics of the 30,827 study participants are summarized in Table 1 by gender (14,383 men and 16,444 women). Among the study population, the total number of those who reported the presence of insomnia was 18.3%, and men and women with insomnia who reported being exposed to noise only were 13.9% and 18.3%, respectively. Additionally, insomnia in those who were exposed to vibration only was 23.9% in males and 26.4% in females. Those who were exposed to both noise and vibration at the same time and reported insomnia were 20.5% and 41.2% in men and women, respectively.

The results of the association between noise and vibration exposure and insomnia are shown in Table 2. The odds ratio [OR] of insomnia among male and female workers who were exposed to noise and/or vibration compared to workers who were not exposed is shown as follows: noise exposure: in men 1.10 (95% CI 0.94–1.28) and in women 1.07 (95% CI 0.91–1.26); vibration exposure: in men 1.84 (95% CI 1.54–2.19) and in women 1.58 (95% CI 1.34–1.86); noise plus vibration exposure: 1.83 (95% CI 1.61–2.07) and 3.14 (95% CI 2.76–3.57) in men and women, respectively.

In Table 3, additional logistic regression analyses were carried out to further investigate the association of insomnia with various occupational variables in workers who were exposed to noise and/or vibration. In men, those who were exposed to vibration and did not wear personal protective equipment when needed (OR 2.67; (95% CI, 1.07–6.67)) as well as those working more than 11 years at their current job (OR 2.14; (95% CI, 1.56–2.94)) had a high risk of insomnia. In addition, working in a business with 10 or fewer employees (OR 2.11; (95% CI, 1.82–2.44)) whilst being exposed to simultaneous noise and vibration exposure had a strong correlation with insomnia as well. In women, those who were exposed to both noise and vibration and did not wear personal protective equipment (PPE) when required (OR 2.07; (95% CI, 1.16–3.70)) and those working at a business with 10 or fewer employees (OR 3.56; (95% CI, 3.10–4.10)) had an increased risk of insomnia.

Table 4 presents the results of subgroup analysis indicating the degree of exposure to noise and/or vibration exposure and the association with insomnia. Both male and female workers showed a dose–response relationship for noise only and vibration only exposure to a certain extent as shown in the following: “Degree of noise exposure” in men gradually increased from 1 to 3 points (OR 1.22 (95% CI, 1.06–1.40); OR 1.67 (95% CI, 1.41–1.98); OR 1.77 (95% CI, 1.43–1.98)) and then decreased from 4 to 5 points (OR 1.57 (95% CI, 1.27–1.94); OR 0.96 (95% CI, 0.71–1.31)). In women, it gradually increased from 1 to 4 points (OR 1.57 (95% CI, 1.36–1.81); OR 2.13 (95% CI, 1.75–2.58); OR 2.86 (95% CI, 2.24–3.65); OR 2.97 (95% CI, 2.29–3.83)) and then decreased at 5 points (OR 1.31 (95% CI, 1.27–1.94)). For “degree of vibration exposure”, a similar trend can be seen in men from 1 to 4 points (OR 1.54 (95% CI, 1.35–1.76); OR 2.10 (95% CI, 1.75–2.53); OR 2.17 (95% CI, 1.74–2.71); OR 2.25 (95% CI, 1.75–2.89)) and decreased at 5 points (OR 1.83 (95% CI, 1.24–2.69). In women, it increased from 1 to 4 points (OR 1.80 (95% CI, 1.57–2.06); OR 2.94 (95% CI, 2.40–3.59); OR 3.74 (95% CI, 2.86–4.88); OR 4.68 (95% CI, 3.49–6.28)) and then decreased at 5 points (OR 1.82 (95% CI, 1.04–3.17)). For “degree of noise plus vibration exposure”, a dose–response relationship could be seen in both males and females. The OR of insomnia increased with increasing degree of exposure by 1 to 3 points, 4 to 5 points, and 6 to 8 points in men with OR 1.44 (95% CI, 1.29–1.62); OR 1.88 (95% CI, 1.58–2.24); and OR 2.05 (95% CI, 1.69–2.48), respectively, and then decreased at 9 to 10 points (OR 1.37 (95% CI, 0.91–2.04)), and in women, it increased with OR 1.57 (95% CI, 1.41–1.74); OR 2.24 (95% CI, 1.83–2.73); and OR 5.25 (95% CI, 4.12–6.67), respectively, and then decreased at 9 to 10 points (OR 2.69 (95% CI, 1.40–5.16)).

Table 5 shows the results of the synergistic effects of simultaneous noise and vibration exposure on insomnia risk. The relative excess risk due to interaction (RERI), attributable proportion (AP), and synergy index (SI) for the combination were −0.10, −0.06, and 0.75 in men and 1.49, 0.47, and 1.60 in women, respectively. In males, none of the values were statistically significant. Therefore, no synergistic effect was seen. However, in females, RERI and AP values were greater than zero and SI exceeded one, and were statistically significant, signifying synergistic interaction.

## 3. Discussion

This study used the 5th Korean Working Conditions Survey (KWCS) to analyze the association between occupational noise and vibration exposure and insomnia. Our findings suggest that more than noise exposure, exposure to vibration had a prominent effect on insomnia. In addition, the prevalence of insomnia was higher in females than in males, which is consistent with previous findings that women are more vulnerable to mental health problems than men [25,26]. However, with the exception of simultaneous noise and vibration exposure, the OR of insomnia was higher in males than females. This could be explained by prior studies reporting that men are more likely to be exposed to hazardous occupational conditions than women, thereby making them more susceptible to their detrimental effects [27,28]. Previous studies indicated that long-term exposure to noise or vibration can endlessly stimulate the autonomic nervous system [29], causing sustained activation of the central autonomic system and induction of sympathetic nervous activity [30]. Insomnia may arise from stimulation of the peripheral nervous system [31,32]. Another study showed that workers exposed to vibration from operating heavy-duty machinery or working inside buildings resulted in an imbalance in the sympathetic nervous system [33]. It was previously revealed that chronic vibration exposure had a significant effect on poor sleep, independent of noise level exposure [33,34].

Prior laboratory research regarding noise exposure effects on sleep has shown ambiguous results. It seems that noise exposure effects are complex, and the absence of a clear dose–effect relationship is due to several factors including noise severity and the individual’s sensitivity. Another study suggested that the effect of noise exposure on insomnia may eventually be habituated, thereby offering another explanation as to why the effect was not as prominently shown in this study [35].

The synergistic effect of noise and vibration has been proven in prior studies on health outcomes such as hearing loss [36,37], headache/eyestrain [7], and cognitive performance [38]. The rationale behind investigating the effect of both noise and vibration with insomnia is that, for instance, when handling large equipment or driving large vehicles, workers are often exposed to noise and vibration simultaneously in their work environments. A prior study carried out in Korea showed that combined noise and vibration exposure had a greater effect on the increased total of nervous system-related disorders, which included headache/eyestrain, fatigue, and sleep disturbance/insomnia [9]. Therefore, there is a need to investigate the combined effect of noise and vibration. Through the results of interaction analysis, the synergistic effect of both combined noise and vibration exposure on insomnia was seen in females, but not in males, supporting prior research [39]. A hypothesis pertaining to the reason why the synergistic effects were not seen in men could be attributed to the healthy worker effect, whereby male workers who were more likely to be exposed to severe hazardous occupational factors could not tolerate working conditions where simultaneous noise and vibration exposure occurred and either quit their jobs or retired.

Regarding the subgroup analysis of occupational variables and noise and vibration exposure in individuals with insomnia, men and women who did not wear PPE when required and were exposed to vibration exposure only or both noise and vibration exposure, therefore eliminating a form of protection against the two exposures, had a significantly higher risk of insomnia supporting the findings of a prior study [9]. The implications of not wearing specialized PPE such as earplugs for noise exposure or anti-vibration gloves for vibration exposure can be severe; therefore, the World Health Organization (WHO) released guidelines regarding PPE use to lessen these harmful effects [40]. Additionally, men and women who worked in workplaces with 10 or fewer employees had a higher risk of insomnia compared to those who worked larger businesses. This could be linked to the previous point in the sense that smaller workplaces are not as fully equipped and prepared for safety protocols against exposure to these occupational hazards including the provision of PPE.

In addition, men who were exposed to vibration and worked more than 11 years had a higher risk of insomnia. This was consistent with a prior study that speculated that chronic exposure to vibration may result in a constant state of hyperarousal of the autonomic nervous system, leading to psychological disorders including disturbed sleep [41].

A dose–response relationship could be inferred from the variable of interest subgroup analysis presenting the relationship of the extent of noise and vibration exposure both separately and combined with insomnia. In noise exposure and vibration exposure, individually and combined, with increasing time spent being exposed to these factors, the risk of insomnia gradually increased, signifying a dose–response relationship. However, when the exposure was 4 or 5 points or, in the case of simultaneous exposure, 9 to 10 points, which indicated exposure to either noise or vibration almost all or all the time, the OR of insomnia suddenly decreased. This could again be explained due to the phenomenon of the healthy worker effect, where those who suffered severe health consequences or could not tolerate constant exposure to noise or vibration quit, retired, or changed occupations.

The following limitations were recognized in our study. First, as the obtained data were analyzed cross-sectionally, a causal relationship could not be verified. Second, this study involved self-reported questionnaires; therefore, we could not rule out the possibility of response and recall bias. Third, information provided in this survey was lacking key variables such as smoking and drinking habits. Fourth, different types of vibration exist, such as whole-body vibration and hand-arm vibration [42], but they were not specified in the survey. Fifth, we could not investigate people who experienced early-morning awakening with the inability to return to sleep, as there was limited information on sleep variables. On the other hand, the most commonly reported symptom of insomnia was reported to be difficulty maintaining sleep followed by difficulty in initiating sleep [43,44]. Finally, there was a lack of objective assessment in regard to both exposure and outcome evaluation. For example, we could not quantify the amount of noise and vibration exposure in the workplace and could not use more reliable measures of assessment of sleep problems, e.g., polysomnography.

On the other hand, our study’s main strong point is that, to the extent of our knowledge, this is the first study in South Korea and one of the few studies worldwide to focus on the simultaneous exposure of noise and vibration effects on insomnia. Other studies have previously investigated the effect of noise and vibration exposure on a variety of mental health problems [9,45]. However, those exposures were investigated individually, not simultaneously. In addition, another strength that sets apart our study is that we used interaction analysis to evaluate the effects of synergistic exposure to noise and vibration on insomnia risk. Although previous studies have previously investigated the synergistic effect of noise and vibration, those studies mainly investigated their effects on hearing loss [46,47]. Another strength lies in the fact that we investigated the effects of noise and vibration exposure on insomnia in a nationally inclusive sample of Korean workers, and it was stratified by sex.

## 4. Methods

Our study used data obtained from the 2017 Korean Working Conditions Survey led by the Korea Occupational Safety and Health Agency (KOSHA). Since the first KWCS survey in 2006, statistical data have been periodically obtained on Korean workers’ health-related characteristics as well as occupational risk factors. A multistage random-sampling approach based on the Population and Housing Census was used in the KWCS and could be considered to be representative of the overall population of Korean workers. The survey data were collected through direct interviews through house visits, the target population being workers aged ≥ 15 years old. In the event where there was more than one eligible employee, the interviewers carried out interviews with those whose birth date was closest to the research date. Information was obtained about each employee’s general characteristics, occupational characteristics, and state of health. All participants provided written informed consent and were guaranteed anonymity. In the 5th edition of the Korean Working Conditions Survey, a total of 50,027 participants were included. After excluding those with missing data or those who failed or refused to respond, a final population of 30,837 people was selected for this study.

In this study, the dependent variable in question was insomnia. The classification used to examine the presence of insomnia was based on the fifth edition of the Diagnostic and Statistical Manual of Mental Disorders (DSM-5) [44]. The diagnostic criteria for insomnia included those who experience one or more of the following symptoms at least three nights per week for at least three months: (1) difficulty initiating sleep, (2) difficulty maintaining sleep, and (3) early-morning awakening with the inability to return to sleep. The KWCS questionnaire included a question that asked, “Over the past 12 months, did you suffer from sleep-related problems?”, and those who answered “daily”, “several times a week”, or “several times a month” in response to at least one of the aforementioned symptoms were determined to have insomnia.

The variable of interest in this study was noise and vibration exposure. Noise and vibration exposure were assessed by the following two questions: “In your workplace, are you exposed to noise so loud that you have to raise your voice to keep a conversation during work?” and “How much are you exposed to hand-transmitted vibration or vibration generated by machinery?” Seven responses were possible depending on the time spent being exposed to the aforementioned ergonometric factors: never, almost never, one-quarter of the time, half of the time, three-quarters of the time, almost all the time, and all of the time. These were then clustered dichotomously, as follows: “never” and “almost never” was reclassified as “no exposure”, and the rest of the responses were grouped into the exposed group for noise, vibration, and noise plus vibration exposure. In the subgroup analysis of our variable of interest, the degree of noise and vibration was each classified using a grade-point system. Zero points were the combined responses of “never” and “almost never”, one point was “one-quarter of the time”, two points was “half of the time”, “three-quarters of the time” was three points, four points was “almost all the time”, and the maximum of five points was given to “all of the time.” Noise plus vibration exposure was the combined total of the noise and vibration exposure degree and was grouped into five categories “zero points”, “one to three points”, “four to five points”, “six to eight points”, and “9 to 10 points”.

Various sociodemographic, health-related, and occupational characteristics were all added as potential confounding variables in this study. Sociodemographic characteristics included the following: gender, age (≤29, 30–39, 40–49, 50–59, ≥60), education level (elementary school degree or lower, middle school degree, high school degree, university degree or higher), and income level per month, which was divided into four quartiles (<150,000, <250,000, <350,000, ≥350,000). Health-related variables encompassed the following: depression, fatigue, presence of hearing problems, presence of headache/eyestrain symptoms, subjective health condition (good, normal, bad), and physical activity in leisure time (every day, several times per week, several times per month, rarely, never). Occupational-related variables included the following: use of PPE including earplugs, helmets, etc., job satisfaction (very satisfied, satisfied, a little unsatisfied, unsatisfied), work and life balance, work duration (≤5 years, 5–10 years, ≥11 years), and working hours per week (≤40 h, 41–50 h, 51–60 h, ≥61 h). Job types were based on the Korean Standard Occupational Classification (6th revision) classified according to three categories: white collar (administrators, professionals, engineers and semi-professionals, and office workers), pink collar (service workers and sales workers), and blue collar (skilled agricultural, forestry, and fishery industry workers; technically skilled worker operators and related skill workers; equipment or machinery operator and assembly workers; and simple laborers) [48]. Other occupational-related variables included shift work, flexible break time, and lastly, size of business (1–9 people, 10–249 people, and ≥250 people).

A chi-squared test was utilized to compare the covariates of the study participants. The association between noise and vibration exposure and insomnia in workers was analyzed via multiple logistic regression, and p-values less than 0.05 were statistically significant. In the subgroup analysis, the association between occupational-related variables and insomnia, as well as the trend for significance between the degree of noise and vibration exposure with insomnia, was carried out and confirmed through p-value for trend analysis. P-values for trend results less than 0.05 were considered statistically significant.

Additive interaction analysis to examine the interaction between noise and vibration exposure and insomnia was carried out. Three measures of additive interactions—RERI, AP, and SI—and their 95% CI were calculated. If RERI and AP did not equal zero and SI exceeded one, then additive interaction was considered present. In addition, if RERI was greater than zero, the interaction was considered synergistic; if RERI was less than zero, an antagonistic interaction was implied. All statistical analyses were conducted using SAS 9.4 software (SAS Inc., Cary, NC, USA).

## 5. Conclusions

In conclusion, our findings suggested an association between workplace noise and vibration exposure and insomnia. From a public health viewpoint, it is important to tackle and address problems affecting the sleep quality of these workers as it negatively impacts workers’ health and quality of life and performance in the workplace.

## Figures and Tables

**Table 1:** General characteristics of study population.

Variables	Insomnia													
			Male (*N* = 14,383)				*p*-Value			Female (*N* = 16,444)				*p*-Value
	*N* = 14,383		Yes		No			*N* = 16,444		Yes		No		
	*N*	%	*N*	%	*N*	%		*N*	%	*N*	%	*N*	%	
**Noise and vibration exposure**							<0.0001							<0.0001
No Exposure	8621	59.9	1274	14.8	7347	85.2		13,028	79.2	2267	17.4	10,761	82.6	
Noise Exposure	1816	12.6	252	13.9	1564	86.1		1191	7.2	218	18.3	973	81.7	
Vibration Exposure	833	5.8	199	23.9	634	76.1		889	5.4	235	26.4	654	73.6	
Noise plus Vibration exposure	3113	21.6	637	20.5	2476	79.5		1336	8.1	551	41.2	785	58.8	
**Age**							<0.0001							<0.0001
≤29	1338	9.3	159	11.9	1179	88.1		1235	7.5	177	14.3	1058	85.7	
30–39	2256	15.7	332	14.7	1924	85.3		1961	11.9	311	15.9	1650	84.1	
40–49	2979	20.7	439	14.7	2540	85.3		3576	21.7	615	17.2	2961	82.8	
50–59	3621	25.2	633	17.5	2988	82.5		4970	30.2	1006	20.2	3964	79.8	
60≤	4189	29.1	799	19.1	3390	80.9		4702	28.6	1162	24.7	3540	75.3	
**Education**							0.0001							<0.0001
Middle school degree	2741	19.1	515	18.8	2226	81.2		4386	26.7	1088	24.8	3298	75.2	
High School degree	6189	43.0	1010	16.3	5179	83.7		7132	43.4	1382	19.4	5750	80.6	
University degree or higher	5453	37.9	837	15.3	4616	84.7		4926	30.0	801	16.3	4125	83.7	
**Income ^a^**							0.0003							0.1309
Q1	2182	15.2	406	18.6	1776	81.4		5540	33.7	1138	20.5	4402	79.5	
Q2	3751	26.1	628	16.7	3123	83.3		6277	38.2	1244	19.8	5033	80.2	
Q3	4248	29.5	698	16.4	3550	83.6		3032	18.4	578	19.1	2454	80.9	
Q4	4202	29.2	630	15.0	3572	85.0		1595	9.7	311	19.5	1284	80.5	
**Depression**							<0.0001							<0.0001
Yes	372	2.6	156	41.9	216	58.1		558	3.4	260	46.6	298	53.4	
No	14,011	97.4	2206	15.7	11,805	84.3		15,886	96.6	3011	19.0	12,875	81.0	
**Fatigue**							<0.0001							<0.0001
Yes	3962	27.5	806	20.3	3156	79.7		4603	28.0	1108	24.1	3495	75.9	
No	10,421	72.5	1556	14.9	8865	85.1		11,841	72.0	2163	18.3	9678	81.7	
**Hearing Problems**							<0.0001							<0.0001
Yes	262	1.8	80	30.5	182	69.5		230	1.4	106	46.1	124	53.9	
No	14,121	98.2	2282	16.2	11,839	83.8		16,214	98.6	3165	19.5	13,049	80.5	
**Headache/Eye strain**							<0.0001							<0.0001
Yes	2002	13.9	475	23.7	1527	76.3		2361	14.4	667	28.3	1694	71.7	
No	12,381	86.1	1887	15.2	10,494	84.8		14,083	85.6	2604	18.5	11,479	81.5	
**Subjective Health Condition**							<0.0001							<0.0001
Good	9538	66.3	1,305	13.7	8233	86.3		9843	59.9	1570	16.0	8273	84.0	
Normal	4208	29.3	856	20.3	3352	79.7		5325	32.4	1215	22.8	4110	77.2	
Bad	637	4.4	201	31.6	436	68.4		1276	7.8	486	38.1	790	61.9	
**Physical Activity in Leisure Time**							0.4184							0.9415
Everyday	326	2.3	64	19.6	262	80.4		268	1.6	65	24.3	203	75.7	
Several times per week	1658	11.5	315	19.0	1343	81.0		1488	9.0	373	25.1	1115	74.9	
Several times per month	3446	24.0	499	14.5	2947	85.5		3151	19.2	560	17.8	2591	82.2	
Rarely	3939	27.4	571	14.5	3368	85.5		4219	25.7	691	16.4	3528	83.6	
Never	5014	34.9	913	18.2	4101	81.8		7318	44.5	1582	21.6	5736	78.4	
**Use of PPE ^b^**							<0.0001							0.1281
Yes	4689	32.6	595	12.7	4094	87.3		3246	19.7	582	17.9	2664	82.1	
No	704	4.9	136	19.3	568	80.7		608	3.7	186	30.6	422	69.4	
Not applicable	8990	62.5	1631	18.1	7359	81.9		12,590	76.6	2503	19.9	10,087	80.1	
**Job Satisfaction**							<0.0001							<0.0001
Yes	10,253	71.3	1418	13.8	8835	86.2		4167	25.3	1255	30.1	2912	69.9	
No	4130	28.7	944	22.9	3186	77.1		12,277	74.7	2016	16.4	10,261	83.6	
**Work and life balance**							<0.0001							<0.0001
Yes	9490	66.0	1432	15.1	8058	84.9		11,387	69.2	2158	19.0	9229	81.0	
No	4893	34.0	930	19.0	3963	81.0		4987	30.3	1113	22.3	3874	77.7	
**Work duration**							0.3461							<0.0001
≤5 years	4887	34.0	787	16.1	4100	83.9		7063	43.0	1234	17.5	5829	82.5	
5-10 years	4367	30.4	715	16.4	3652	83.6		4922	29.9	976	19.8	3946	80.2	
≥11 years	5130	35.7	861	16.8	4269	83.2		4459	27.1	1061	23.8	3398	76.2	
**Working hours/week**							0.1910							<0.0001
≤40 h	2412	16.8	463	19.2	1949	80.8		5143	31.3	1122	21.8	4021	78.2	
41–50 h	6042	42.0	933	15.4	5109	84.6		5704	34.7	1131	19.8	4573	80.2	
51–60 h	3913	27.2	614	15.7	3299	84.3		4000	24.3	704	17.6	3296	82.4	
≥61 h	2016	14.0	352	17.5	1664	82.5		1597	9.7	314	19.7	1283	80.3	
**Job Collar ^c^**							0.0919							0.4592
White	2518	17.5	365	14.5	2153	85.5		2895	17.6	456	15.8	2439	84.2	
Blue	7902	54.9	1345	17.0	6557	83.0		4872	29.6	1180	24.2	3692	75.8	
Pink	3963	27.6	652	16.5	3311	83.5		8677	52.8	1635	18.8	7042	81.2	
**Shift Work**							0.0204							0.5379
Yes	1478	10.3	274	18.5	1204	81.5		1151	7.0	237	20.6	914	79.4	
No	12,905	89.7	2088	16.2	10,817	83.8		15,293	93.0	3034	19.8	12,259	80.2	
**Flexible break time**							0.0706							<0.0001
Yes	11,140	77.5	1863	16.7	9277	83.3		12,255	74.5	2599	21.2	9656	78.8	
No	3243	22.5	499	15.4	2744	84.6		4189	25.5	672	16.0	3517	84.0	
**Size of Business**							0.0038							0.0671
1–9 people	10,416	72.4	1767	17.0	8649	83.0		13,199	80.3	2668	20.2	10,531	79.8	
10–249 people	3467	24.1	525	15.1	2942	84.9		2976	18.1	549	18.4	2427	81.6	
≥250 people	500	3.5	70	14.0	430	86.0		269	1.6	54	20.1	215	79.9	

^a^ income level per month, which was divided into four quartiles (<150,000; <250,000; <350,000, ≥350,000); ^b^ Personal Protective Equipment; ^c^ Job collar types classified according the Korean Standard Occupational Classification.

**Table 2:** Association of Noise & Vibration exposure with Insomnia.

Variables	Insomnia			
	Male		Female	
	Adjusted OR	95% CI	Adjusted OR	95% CI
**Noise and vibration exposure**				
No Exposure	1	-	1	-
Noise Exposure	1.1	(0.94–1.28)	1.07	(0.91–1.26)
Vibration Exposure	1.84	(1.54–2.19)	1.58	(1.34–1.86)
Noise plus Vibration exposure	1.83	(1.61–2.07)	3.14	(2.76–3.57)
**Age**				
≤29	1	-	1	-
30–39	1.28	(1.03–1.59)	1.07	(0.87–1.32)
40–49	1.26	(1.01–1.57)	1.13	(0.93–1.37)
50–59	1.57	(1.26–1.95)	1.23	(1.01–1.49)
60≤	1.68	(1.35–2.11)	1.36	(1.09–1.70)
**Education**				
Middle school degree	1	-	1	-
High School degree	1.16	(1.00–1.35)	1.02	(0.89–1.18)
University degree or higher	1.31	(1.10–1.57)	0.99	(0.83–1.18)
**Income ^a^**				
Q1	0.98	(0.81–1.18)	0.71	(0.60–0.85)
Q2	1.01	(0.88–1.17)	1.01	(0.87–1.17)
Q3	1.11	(0.98–1.26)	1.03	(0.88–1.21)
Q4	1	-	1	-
**Depression**				
Yes	2.44	(1.94–3.06)	2.49	(2.06–3.00)
No	1	-	1	-
**Fatigue**				
Yes	1.06	(0.95–1.19)	0.94	(0.86–1.04)
No	1	-	1	-
**Hearing Problems**				
Yes	1.49	(1.12–1.99)	2.03	(1.52–2.72)
No	1	-	1	-
**Headache/Eye strain**				
Yes	1.28	(1.12–1.45)	1.22	(1.09–1.37)
No	1	-	1	-
**Subjective Health Condition**				
Good	1	-	1	-
Normal	1.43	(1.29–1.59)	1.39	(1.27–1.53)
Bad	2.09	(1.71–2.57)	2.22	(1.90–2.59)
**Physical Activity in Leisure Time**				
Everyday	1	-	1	-
Several times per week	1.03	(0.76–1.40)	1.04	(0.76–1.42)
Several times per month	0.74	(0.55–1.00)	0.72	(0.53–0.97)
Rarely	0.67	(0.50–0.91)	0.55	(0.41–0.75)
Never	0.82	(0.61–1.10)	0.69	(0.51–0.93)
**Use of PPE ^b^**				
Yes	1	-	1	-
No	1.39	(1.10–1.72)	1.48	(1.19–1.83)
Not applicable	1.86	(1.65–2.08)	1.33	(1.19–1.48)
**Job Satisfaction**				
Yes	1	-	1	-
No	1.53	(1.38–1.69)	1.73	(1.59–1.90)
**Work and life balance**				
Yes	1	-	1	-
No	1.16	(1.05–1.28)	1.15	(1.04–1.26)
**Work duration**				
≤5 years	1	-	1	-
5–10 years	0.96	(0.85–1.08)	1.06	(0.95–1.17)
≥11 years	0.86	(0.75–0.97)	1.03	(0.92–1.16)
**Working hours/week**				
≤40 h	1	-	1	-
41–50 h	0.83	(0.72–0.96)	0.9	(0.81–1.01)
51–60 h	0.78	(0.67–0.91)	0.68	(0.59–0.77)
≥61 h	0.75	(0.63–0.90)	0.64	(0.54–0.76)
**Job Collar ^c^**				
White	1	-	1	-
Blue	1.01	(0.87–1.19)	1.1	(0.93–1.29)
Pink	1.11	(0.95–1.30)	1.12	(0.98–1.29)
**Shift Work**				
Yes	1.22	(1.04–1.42)	1.19	(1.02–1.40)
No	1	-	1	-
**Flexible break time**				
Yes	1	-	1	-
No	0.87	(0.78–0.98)	0.73	(0.66–0.81)
**Size of Business**				
1–9 people	1.1	(0.84–1.45)	0.89	(0.65–1.22)
10–249 people	0.99	(0.75–1.31)	0.89	(0.64–1.23)
≥250 people	1	-	1	-

^a^ income level per month, which was divided into four quartiles (<150,000; <250,000; <350,000, ≥350,000); ^b^ Personal Protective Equipment; ^c^ Job collar types classified according the Korean Standard Occupational Classification.

**Table 3:** The results of subgroup analysis stratified by occupational–related characteristics.

Variables	Insomnia							
	Noise and Vibration Exposure							
	No	Noise Exposure		Vibration Exposure		Noise plus Vibration Exposure		*p*-Value for Trend
	Adjusted OR	Adjusted OR	95% CI	Adjusted OR	95% CI	Adjusted OR	95% CI	
**Male**								
**Use of PPE ^a^**								
Yes	1	1.1	(0.85–1.44)	1.24	(0.83–1.86)	1.32	(1.06–1.66)	0.09
No	1	1.2	(0.70–2.07)	2.67	(1.07–6.67)	1.14	(0.68–1.92)	0.075
Not applicable	1	0.95	(0.76–1.18)	1.97	(1.61–2.41)	2.5	(2.13–2.95)	<0.0001
**Job Satisfaction**								
Yes	1	0.99	(0.81–1.20)	2.07	(1.66–2.58)	1.59	(1.34–1.88)	<0.0001
No	1	1.26	(0.96–1.64)	1.55	(1.15–2.09)	2.13	(1.75–2.59)	<0.0001
**Work and life balance**								
Yes	1	1.09	(0.90–1.33)	1.98	(1.57–2.50)	1.8	(1.53–2.12)	<0.0001
No	1	1.1	(0.84–1.42)	1.64	(1.25–2.15)	1.84	(1.50–2.26)	<0.0001
**Work duration**								
≤5 years	1	1.13	(0.82–1.56)	1.87	(1.40–2.49)	2.08	(1.66–2.61)	<0.0001
5–10 years	1	1.21	(0.90–1.63)	1.62	(1.17–2.24)	2.16	(1.71–2.74)	<0.0001
≥11 years	1	0.99	(0.79–1.25)	2.14	(1.56–2.94)	1.43	(1.16–1.75)	<0.0001
**Working hours/week**								
≤40 h	1	1.45	(1.00–2.12)	2.87	(1.86–4.41)	3.46	(2.59–4.62)	<0.0001
41–50 h	1	1.04	(0.80–1.35)	1.62	(1.20–2.18)	1.6	(1.30–1.97)	0.0001
51–60 h	1	1	(0.74–1.34)	1.7	(1.22–2.37)	1.41	(1.09–1.82)	0.0014
≥61 h	1	1.01	(0.68–1.51)	1.94	(1.25–3.00)	1.57	(1.11–2.22)	0.0019
**Job Collar ^b^**								
White	1	1.22	(0.72–2.07)	2.04	(1.33–3.12)	1.72	(1.17–2.51)	0.0002
Blue	1	1.07	(0.89–1.29)	1.93	(1.48–2.51)	1.76	(1.51–2.05)	<0.0001
Pink	1	1.05	(0.72–1.53)	1.79	(1.33–2.40)	2.58	(1.92–3.48)	<0.0001
**Shift Work**								
Yes	1	1.54	(0.90–2.62)	1.66	(1.04–2.64)	1.44	(0.94–2.19)	0.0141
No	1	1.07	(0.90–1.26)	1.86	(1.54–2.26)	1.86	(1.62–2.12)	<0.0001
**Flexible break time**								
Yes	1	1.1	(0.92–1.31)	2.07	(1.70–2.53)	2.03	(1.76–2.34)	<0.0001
No	1	1.09	(0.77–1.55)	1.15	(0.78–1.71)	1.21	(0.90–1.61)	0.3698
**Size of Business**								
1–9 people	1	1.15	(0.96–1.37)	1.89	(1.54–2.34)	2.11	(1.82–2.44)	<0.0001
10–249 people	1	1.03	(0.71–1.50)	1.76	(1.25–2.49)	1.26	(0.96–1.66)	0.0019
≥250 people	1	0.35	(0.09–1.33)	1.38	(0.37–5.14)	1.31	(0.59–2.92)	0.6837
**Female**								
**Use of PPE ^a^**								
Yes	1	0.98	(0.74–1.31)	1.33	(0.92–1.93)	1.54	(1.17–2.02)	0.0469
No	1	0.99	(0.51–1.92)	1.37	(0.54–3.48)	2.07	(1.16–3.70)	0.0534
Not applicable	1	1.1	(0.89–1.36)	1.69	(1.40–2.03)	4.45	(3.80–5.21)	<0.0001
**Job Satisfaction**								
Yes	1	1.09	(0.89–1.33)	1.71	(1.39–2.11)	3.01	(2.54–3.55)	<0.0001
No	1	1.02	(0.76–1.36)	1.39	(1.07–1.81)	3.15	(2.57–3.87)	<0.0001
**Work and life balance**								
Yes	1	1.09	(0.89–1.33)	1.58	(1.28–1.96)	3.16	(2.69–3.72)	<0.0001
No	1	1.07	(0.81–1.42)	1.62	(1.25–2.10)	3.05	(2.46–3.77)	<0.0001
**Work duration**								
≤5 years	1	1.02	(0.75–1.40)	1.59	(1.24–2.04)	3.79	(3.07–4.67)	<0.0001
5–10 years	1	0.94	(0.67–1.30)	1.92	(1.46–2.54)	3.28	(2.56–4.20)	<0.0001
≥11 years	1	1.17	(0.92–1.49)	1.24	(0.87–1.78)	2.54	(2.03–3.17)	<0.0001
**Working hours/week**								
≤40 h	1	1.17	(0.86–1.59)	1.39	(1.03–1.87)	5.77	(4.58–7.27)	<0.0001
41–50 h	1	1.25	(0.93–1.67)	1.83	(1.37–2.44)	2.37	(1.87–2.99)	<0.0001
51–60 h	1	0.85	(0.61–1.19)	2.01	(1.46–2.76)	2.11	(1.59–2.78)	<0.0001
≥61 h	1	0.97	(0.60–1.57)	1.01	(0.60–1.71)	3.16	(1.99–5.03)	0.1118
**Job Collar ^b^**								
White	1	1.16	(0.64–2.09)	2.72	(1.85–4.02)	2.92	(1.89–4.50)	<0.0001
Blue	1	1.09	(0.87–1.36)	1.28	(0.92–1.80)	2.93	(2.43–3.54)	<0.0001
Pink	1	1.07	(0.81–1.41)	1.56	(1.26–1.94)	3.76	(3.07–4.60)	<0.0001
**Shift Work**								
Yes	1	0.94	(0.42–2.15)	1.44	(0.85–2.44)	1.48	(0.83–2.66)	0.2207
No	1	1.09	(0.92–1.29)	1.6	(1.35–1.90)	3.25	(2.85–3.71)	<0.0001
**Flexible break time**	1							
Yes	1	1.09	(0.91–1.30)	1.59	(1.32–1.92)	3.67	(3.18–4.24)	<0.0001
No	1	0.98	(0.67–1.44)	1.53	(1.10–2.12)	1.64	(1.20–2.26)	0.0021
**Size of Business**								
1–9 people	1	1.06	(0.89–1.27)	1.62	(1.35–1.95)	3.56	(3.10–4.10)	<0.0001
10–249 people	1	1.34	(0.84–2.13)	1.52	(1.04–2.22)	1.82	(1.29–2.59)	0.0019
≥250 people	1	0.23	(0.03–1.68)	1.16	(0.28–4.89)	0.42	(0.10–1.69)	0.5928

Adjusted for other covariates; ^a^ Personal Protective Equipment; ^b^ Job collar types classified according the Korean Standard Occupational Classification.

**Table 4:** The results of degree of noise and/or vibration exposure with insomnia.

Variables	Insomnia					
	Male		*p*-Value for Trend	Female		*p*-Value for Trend
	Adjusted OR	95% CI		Adjusted OR	95% CI	
**Degree of Noise Exposure ^a^**			<0.0001			<0.0001
0	1	-		1	-	
1	1.22	(1.06–1.40)		1.57	(1.36–1.81)	
2	1.67	(1.41–1.98)		2.13	(1.75–2.58)	
3	1.77	(1.43–2.21)		2.86	(2.24–3.65)	
4	1.57	(1.27–1.94)		2.97	(2.29–3.83)	
5	0.96	(0.71–1.31)		1.31	(0.84–2.04)	
**Degree of Vibration Exposure ^a^**			<0.0001			<0.0001
0	1	–		1	–	
1	1.54	(1.35–1.76)		1.8	(1.57–2.06)	
2	2.1	(1.75–2.53)		2.94	(2.40–3.59)	
3	2.17	(1.74–2.71)		3.74	(2.86–4.88)	
4	2.25	(1.75–2.89)		4.68	(3.49–6.28)	
5	1.83	(1.24–2.69)		1.82	(1.04–3.17)	
**Degree of Vibration+ Noise Exposure ^b^**			<0.0001			<0.0001
0	1	–		1	–	
1 to 3	1.44	(1.29–1.62)		1.57	(1.41–1.74)	
4 to 5	1.88	(1.58–2.24)		2.24	(1.83–2.73)	
6 to 8	2.05	(1.69–2.48)		5.25	(4.12–6.67)	
9 to 10	1.37	(0.91–2.04)		2.69	(1.40–5.16)	

^a^ Both degree of noise exposure only and vibration exposure only were assigned to a grade point scale with 0 points being; no exposure at all and 5 points being the maximum indicating exposure all the time; ^b^ The total combined points of noise exposure and vibration exposure with 0 points being the minimum and 10 points being the maximum total degree of exposure.

**Table 5:** Additive interaction of noise and vibration exposure on insomnia.

Additive Interaction	Insomnia	
	Noise plus Vibration Exposure	
	Adjusted OR	95% CI
**Male**		
**RERI ^a^**	−0.1	(−0.49–0.28)
**AP ^b^**	−0.06	(−0.44–0.33)
**Synergy Index**	0.75	(0.37–1.14)
**Female**		
**RERI ^a^**	1.49	(1.02–1.96)
**AP ^b^**	0.47	(0.00–0.94)
**Synergy Index**	1.6	(1.13–2.07)

Adjusted for other covariates; ^a^ Relative excess risk due to interaction; ^b^ Attributable proportion.

## References

[1] Pyykko I., Farkkila M., Inaba R., Starck J., Pekkarinen J. (1994). Effect of hand-arm vibration on inner ear and cardiac functions in man. Nagoya J. Med. Sci..

[2] Erdogan E., Yazgan M.E. (2009). Landscaping in reducing traffic noise problem in cities: Ankara case. Afr. J. Agric. Res..

[3] Takeuchi T., Futatsuka M., Imanishi H., Yamada S. (1986). Pathological changes observed in the finger biopsy of patients with vibration-induced white finger. Scand. J. Work Environ. Health.

[4] Lang T., Fouriaud C., Jacquinet-Salord M.-C. (1992). Length of occupational noise exposure and blood pressure. Int. Arch. Occup. Environ. Health.

[5] Ising H., Kruppa B. (2003). Health effects caused by noise: Evidence in the literature from the past 25 years. Noise Health.

[6] Passchier-Vermeer W., Passchier W.F. (2000). Noise exposure and public health. Environ. Health Perspect..

[7] Kim J., Lee W., Won J.-U., Yoon J.-H., Seok H., Kim Y.-K., Lee S., Roh J. (2017). The relationship between occupational noise and vibration exposure and headache/eyestrain, based on the fourth Korean Working Condition Survey (KWCS). PLoS ONE.

[8] Jiao K., Li Z., Chen M., Wang C., Qi S. (2004). Effect of different vibration frequencies on heart rate variability and driving fatigue in healthy drivers. Int. Arch. Occup. Environ. Health.

[9] Lee S., Lee W., Roh J., Won J.-U., Yoon J.-H. (2017). Symptoms of Nervous System Related Disorders Among Workers Exposed to Occupational Noise and Vibration in Korea. J. Occup. Environ. Med..

[10] Stansfeld S.A., Matheson M.P. (2003). Noise pollution: Non-auditory effects on health. Br. Med Bull..

[11] Evandt J., Oftedal B., Hjertager Krog N., Nafstad P., Schwarze P.E., Marit Aasvang G. (2016). A Population-Based Study on Nighttime Road Traffic Noise and Insomnia. Sleep.

[12] Muzet A. (2007). Environmental noise, sleep and health. Sleep Med. Rev..

[13] Halperin D. (2014). Environmental noise and sleep disturbances: A threat to health?. Sleep Sci..

[14] Ohayon M.M. (2002). Epidemiology of insomnia: What we know and what we still need to learn. Sleep Med. Rev..

[15] Miedema H.M.E., Vos H. (2007). Associations between Self-Reported Sleep Disturbance and Environmental Noise Based on Reanalyses of Pooled Data from 24 Studies. Behav. Sleep Med..

[16] Bonnet M.H., Arand D.L. (2003). Clinical effects of sleep fragmentation versus sleep deprivation. Sleep Med. Rev..

[17] Martin S.E., Engleman H.M., Deary I.J., Douglas N.J. (1996). The effect of sleep fragmentation on daytime function. Am. J. Respir. Crit. Care Med..

[18] Granö N., Vahtera J., Virtanen M., Keltikangas-Järvinen L., Kivimäki M. (2008). Association of hostility with sleep duration and sleep disturbances in an employee population. Int. J. Behav. Med..

[19] Daley M., Morin C.M., LeBlanc M., Grégoire J.P., Savard J., Baillargeon L. (2009). Insomnia and its relationship to health-care utilization, work absenteeism, productivity and accidents. Sleep Med..

[20] Léger D., Bayon V., Ohayon M.M., Philip P., Ement P., Metlaine A., Chennaoui M., Faraut B. (2014). Insomnia and accidents: Cross-sectional study (EQUINOX) on sleep-related home, work and car accidents in 5293 subjects with insomnia from 10 countries. J. Sleep Res..

[21] Berglund B., Lindvall T., Schwela D.H., World Health Organization (1999). Guidelines for Community Noise.

[22] Seong J.C., Park T.H., Ko J.H., Chang S.I., Kim M., Holt J.B., Mehdi M.R. (2011). Modeling of road traffic noise and estimated human exposure in Fulton County, Georgia, USA. Environ. Int..

[23] Lee H.-S., Choi S.-B. (2000). Control and Response Characteristics of a Magneto-Rheological Fluid Damper for Passenger Vehicles. J. Intell. Mater. Syst. Struct..

[24] Griffin M.J. (1997). Measurement, evaluation, and assessment of occupational exposures to hand-transmitted vibration. Occup. Environ. Med..

[25] La Y.K., Choi Y.H., Chu M.K., Nam J.M., Choi Y.-C., Kim W.-J. (2020). Gender differences influence over insomnia in Korean population: A cross-sectional study. PLoS ONE.

[26] Kim S.M., Jung J.-W., Park I.-W., Ahn C.M., Kim Y.-I., Yoo K.-H., Chun E.M., Jung J.Y., Park Y.S., Park J.H. (2016). Gender Differences in Relations of Smoking Status, Depression, and Suicidality in Korea: Findings from the Korea National Health and Nutrition Examination Survey 2008–2012. Psychiatry Investig..

[27] Lee W., Lee J.-G., Yoon J.-H., Lee J.-H. (2020). Relationship between occupational dust exposure levels and mental health symptoms among Korean workers. PLoS ONE.

[28] Park S.J., Jung M., Sung J.H. (2019). Influence of Physical and Musculoskeletal Factors on Occupational Injuries and Accidents in Korean Workers Based on Gender and Company Size. Int. J. Environ. Res. Public Health.

[29] Seidman M.D., Standring R.T. (2010). Noise and quality of life. Int. J. Environ. Res. Public Health.

[30] Yoon J.-H., Won J.-U., Lee W., Jung P.K., Roh J. (2014). Occupational noise annoyance linked to depressive symptoms and suicidal ideation: A result from nationwide survey of Korea. PLoS ONE.

[31] Hardoy M.C., Carta M.G., Marci A.R., Carbone F., Cadeddu M., Kovess V., Dell’Osso L., Carpiniello B. (2005). Exposure to aircraft noise and risk of psychiatric disorders: The Elmas survey—Aircraft noise and psychiatric disorders. Soc. Psychiatry Psychiatr. Epidemiol..

[32] Murata K., Araki S., Maeda K. (1991). Autonomic and peripheral nervous system dysfunction in workers exposed to hand-arm vibration: A study of R-R interval variability and distribution of nerve conduction velocities. Int. Arch. Occup. Environ. Health.

[33] Harada N. (1994). Autonomic nervous function of hand-arm vibration syndrome patients. Nagoya J. Med. Sci..

[34] Smith M.G., Croy I., Ögren M., Persson Waye K. (2013). On the Influence of Freight Trains on Humans: A Laboratory Investigation of the Impact of Nocturnal Low Frequency Vibration and Noise on Sleep and Heart Rate. PLoS ONE.

[35] Griefahn B. (2002). Sleep disturbances related to environmental noise. Noise Health.

[36] Zhu S.-K., Sakakibara H., Yamada S.Y. (1997). Combined effects of hand-arm vibration and noise on temporary threshold shifts of hearing in healthy subjects. Int. Arch. Occup. Environ. Health.

[37] Pekkarinen J. (1995). Noise, impulse noise, and other physical factors: Combined effects on hearing. Occup. Med..

[38] Ljungberg J., Neely G., Lundström R. (2004). Cognitive performance and subjective experience during combined exposures to whole-body vibration and noise. Int. Arch. Occup. Environ. Health.

[39] Arnberg P.W., Bennerhult O., Eberhardt J.L. (1990). Sleep disturbances caused by vibrations from heavy road traffic. J. Acoust. Soc. Am..

[40] Goelzer B.H.C., Sehrndt G. Occupational Exposure to Noise: Evaluation, Prevention and Control. https://www.who.int/occupational_health/publications/occupnoise/en/.

[41] Seidel H. (1993). Selected health risks caused by long-term, whole-body vibration. Am. J. Ind. Med..

[42] Kim J.-Y., Shin J.-S., Lim M.-S., Choi H.-G., Kim S.-K., Kang H.-T., Koh S.B., Oh S.S. (2018). Relationship between simultaneous exposure to ergonomic risk factors and work-related lower back pain: A cross-sectional study based on the fourth Korean working conditions survey. Ann. Occup. Environ. Med..

[43] Kim S., Jeong W., Kim Y.K., Jang S.-I., Park E.-C. (2019). Gender Differences With Regard to Perceived Job Insecurity and Insomnia in a Working Population. J. Occup. Environ. Med..

[44] American Psychiatric Association (2013). Diagnostic and Statistical Manual of Mental Disorders: DSM-5.

[45] Jung S.W., Lee J.-H., Lee K.-J., Kim H.-R. (2019). Association between Occupational Physicochemical Exposures and Headache/Eyestrain Symptoms among Korean Indoor/Outdoor Construction Workers. Saf. Health Work.

[46] Boettcher F.A., Henderson D., Gratton M.A., Danielson R.W., Byrne C.D. (1987). Synergistic Interactions of Noise and Other Ototraumatic Agents. Ear Hear..

[47] Sisto R., Botti T., Cerini L., Di Giovanni R., Marchetti E., Lunghi A., Sacco F., Sanjust F., Tirabasso A., Moleti A. (2017). Synergistic effects of noise and hand-arm vibration on distortion product otoacoustic emissions in healthy subjects. Int. J. Ind. Ergon..

[48] Choi S.B., Yoon J.-H., Lee W. (2019). The Modified International Standard Classification of Occupations defined by the clustering of occupational characteristics in the Korean Working Conditions Survey. Ind. Health.

